# Identification of predictors for mini-mental state examination and revised Hasegawa's Dementia Scale scores using MR-based brain morphometry

**DOI:** 10.1016/j.ejro.2021.100359

**Published:** 2021-05-24

**Authors:** Koji Yamashita, Takahiro Kuwashiro, Kensuke Ishikawa, Kiyomi Furuya, Shino Harada, Seitaro Shin, Noriaki Wada, Chika Hirakawa, Yasushi Okada, Tomoyuki Noguchi

**Affiliations:** a Department of Radiology, Clinical Research Institute, National Hospital Organization Kyushu Medical Center, 1-8-1 Jigyohama, Chuo-ku, Fukuoka, 810-0065, Japan; b Department of Cerebrovascular Medicine and Neurology, Clinical Research Institute, National Hospital Organization Kyushu Medical Center, 1-8-1 Jigyohama, Chuo-ku, Fukuoka, 810-0065, Japan; c Department of Psychiatry, Clinical Research Institute, National Hospital Organization Kyushu Medical Center, 1-8-1 Jigyohama, Chuo-ku, Fukuoka, 810-0065, Japan; d Department of Medical Technology, Division of Radiology, National Hospital Organization Kyushu Medical Center, 1-8-1 Jigyohama, Chuo-ku, Fukuoka, 810-0065, Japan

**Keywords:** 3D, 3-dimensional, AD, Alzheimer’s disease, ApoE, apolipoprotein E, ERC, entorhinal cortex, GM, gray matter, HDS-R, revised Hasegawa's Dementia Scale, MMSE, Mini-Mental State Examination, MPRAGE, magnetization-prepared rapid gradient-echo, VOI, voxel of interest, VSRAD, Voxel-based specific regional analysis system for Alzheimer’s disease, WM, white matter, Magnetic resonance imaging, Cerebral cortex, Mini-Mental State examination

## Abstract

**Purpose:**

The early detection of cognitive function decline is crucial to help manage or slow the progression of symptoms. The Mini-Mental State Examination (MMSE) and revised Hasegawa's Dementia Scale (HDS-R) are widely used in screening for cognitive impairment. The purpose of this study was to explore common predictors of the two different cognitive testing systems using MR-based brain morphometry.

**Materials and Methods:**

This retrospective study included 200 subjects with clinical suspicion of cognitive impairment who underwent 3D T1-weighted MRI at our institution between February 2019 and August 2020. Variables related to the volume of deep gray matter and 70 cortical thicknesses were obtained from the MR images using voxel-based specific regional analysis system for Alzheimer’s disease (VSRAD) and FreeSurfer software. The correlation between each variable including age and MMSE/HDS-R scores was evaluated using uni- and multi-variate logistic regression analyses.

**Results:**

In univariate analysis, parameters include hippocampal volume and bilateral entorhinal cortex (ERC) thickness showed moderate correlation coefficients with both MMSE and HDS-R scores. Multivariate analysis demonstrated the right ERC thickness was the common parameter which significantly correlates with both MMSE and HDS-R scores (p < 0.05).

**Conclusion:**

Right ERC thickness appears to offer a useful predictive biomarker for both MMSE and HDS-R scores.

## 1 Introduction

Assessment of cognitive function in the elderly represents an essential step toward the detection of early-stage dementia. Subsequent early intervention could slow the progression of the disease. The Mini-Mental State Examination (MMSE) and revised Hasegawa's Dementia Scale (HDS-R) are commonly used in screening for cognitive impairment [1]. MMSE reportedly provides reliable scores with no influence from repetition or learning, and consistent results from repeat testing by the same or different examiners [2]. HDS-R has been used in East-Asian countries [3,4] and may offer advantages over MMSE in that the scale could be administered to patients with motor impairment [3]. Nevertheless, MMSE and HDS-R have complementary roles for detecting cognitive impairment in routine clinical practice.

Magnetic resonance imaging (MRI) is the most commonly employed non-invasive technique for visualization of brain structures with high reproducibility. MRI is playing an increasingly important role in morphological evaluation, especially since the advent of high-spec analytical tools. This has been shown by the relationship between cortical thickness and cognitive test performance in healthy individuals irrespective of scan sessions, scanners, and field strengths [5,6]. Atrophy of the hippocampus and entorhinal cortex (ERC) has been shown using MRI even in the preclinical stage of Alzheimer’s disease (AD) [7,8].

Voxel-based specific regional analysis system for Alzheimer’s disease (VSRAD) is software proposed by Matsuda, based on voxel-based morphometry. VSRAD has been applied in routine clinical practice to diagnose and monitor AD [9]. FreeSurfer is freely available software that is used around the world. The analysis contains surface-based (cortical thickness) and voxel-based (volume) results [10,11]. We hypothesized that the ERC, hippocampus, or both would play a pivotal role in cognitive function. In other words, atrophy of the ERC or hippocampus based on voxel- and surface-based analyses should be associated with declines in MMSE and HDS-R scores.

This study aimed to explore common predictors of both MMSE and HDS-R scores using MR-based brain morphometry.

## 2 Materials and methods

### 2.1 Subjects

This retrospective study was approved by the institutional review board of our hospital for human research. The requirement for informed consent for study participation was waived because of the retrospective design. Our study investigated 200 consecutive subjects (90 men, 110 women; median age, 77 years; range, 36–95 years) with clinically suspected cognitive impairment who underwent 3-dimensional (3D) T1-weighted MRI at our institution between February 2019 and August 2020. Among these, subjects who undertook the MMSE (Japanese version), HDS-R, or both were included in the present study. Exclusion criteria were as follows: i) patients with severe head motion artifacts; or ii) patients with treatable dementias such as idiopathic normal-pressure hydrocephalus, brain tumor, metabolic, infectious, inflammatory, or drug-induced cognitive impairment, or history of stroke other than small vessel disease.

### 2.2 MRI

A 1.5-T MRI unit (Magnetom Symphony Tim; Siemens, Erlangen, Germany) with a 6-channel head coil was used to obtain 3D T1-weighted images. A magnetization-prepared rapid gradient-echo (MPRAGE) sequence was used for 3D T1-weighted imaging with the following parameters: repetition time, 1700 ms; echo time, 3.4 ms; inversion time, 800 ms; flip angle, 15°; sagittal sections, 144; field of view, 230 × 230 mm^2^; and matrix, 256 × 256.

### 2.3 Preprocessing 3D T1-weighted MRI using the FreeSurfer pipeline

All 3D T1-weighted images were preprocessed using the FreeSurfer pipeline version 7.0.0 (http://surfer.nmr.mgh.harvard.edu/) [10,11]. The automatic reconstruction steps contain motion correction, non-uniform intensity normalization, Talairach transform computation, intensity normalization, skull stripping, automatic segmentation of each voxel in the normalized brain into gray matter (GM)/white matter (WM)/cerebrospinal fluid, linear volumetric registration, non-linear volumetric registration, removal of the neck, linear transform array with skull, volumetric labeling and statistics, WM segmentation, removal of the midbrain, tessellation (creating original surface), original surface smoothing, inflation (to minimize metric distortion), automatic topology fixing, creation of WM and pial surfaces based on WM-GM and GM-cerebrospinal fluid intensity gradients, creation of binary volume masks, spherical inflation, spherical registration, resampling of the average curvature from atlas to subject, cortical parcellation using the Desikan-Killiany atlas [12], and parcellation statistics (https://surfer.nmr.mgh.harvard.edu/fswiki/recon-all). Volume data of bilateral hippocampi, amygdalas, thalami, putamina, caudate nuclei, globi pallidi, nuclei accumbentes, and total corpora callosa were collected from the cortical parcellation atlas. Mean thicknesses of the right and left hemispheres, and 70 cortical thicknesses (35 variables in each hemisphere) were obtained from the atlas. Total hippocampal volume and the ratio of hippocampus to cerebral volume were also calculated. These variables including subject age were used for further analyses.

### 2.4 Preprocessing 3D T1-weighted MRI using VSRAD software

All MRI data were also analyzed using VSRAD software (VSRAD Advance 2; Eisai, Tokyo, Japan). Based on target voxels of interest (VOIs) derived from VSRAD software, Z-score, percentage of atrophy area, and ratio of VOI to cerebrum were obtained and used for further analyses.

### 2.5 MMSE and HDS-R

To test whether some variables derived from morphometric data correlated with MMSE or HDS-R scores, patients who completed the MMSE or HDS-R at our institution were included for analysis. All subjects who completed the MMSE or HDS-R underwent MRI the same day. All assessments of MMSE and HDS-R were performed by experienced examiners.

### 2.6 Statistical analysis

All statistical analyses were performed using PASW Statistics version 18 (SPSS Inc., Chicago, IL) and graphical plotting was performed in GraphPad Prism 7 (GraphPad Software, La Jolla, San Jose, CA). The correlation between each variable including subject age and MMSE/HDS-R scores were evaluated using Pearson’s correlation coefficient. Subsequently, parameters with moderate correlation coefficients (r > 0.3) from MMSE or HDS-R were determined as independent variables [13] and evaluated using multivariate logistic regression with simultaneous entry.

Values of p < 0.05 were considered indicative of statistical significance in all statistical analyses.

### 2.7 Results

MMSE was performed by 112 patients (49 men, 63 women; median age, 77 years; range, 46–94 years) and HDS-R by 98 patients (54 men, 44 women; median age, 77 years; range, 46–94 years). Median scores were 26 (range, 9–30) for MMSE and 24 (range, 4–30) for HDS-R. Both MMSE and HDS-R were completed by 95 patients, while 17 patients completed MMSE only and 3 completed HDS-R only. Demographic and clinical information about subject samples are presented in Table 1.

**Table 1:** Summary of Demographic and Clinical Characteristics.

Characteristic	(N = 200) MMSE (n = 112)	HDS-R (n =98)
M/F	49/63	54/44
Age (yo, median)	46 – 94 (77)	46 – 94 (77)
Score(median)	9 – 30(26)	4 – 30 (24)

### 2.8 Univariate analysis between MMSE and variables

Volumes of the right, left and total hippocampus, right and left amygdala, and right caudate nucleus, ratio of hippocampus to cerebral volume, thicknesses of the right and left ERC, right and left insula, right and left temporal pole, right superior temporal lobe, right lateral occipital lobe, left inferior temporal lobe, left superior temporal lobe, and right superior parietal lobe from FreeSurfer, and Z-score, percentage atrophy area in the VOI from VSRAD, and subject ages correlated significantly with MMSE scores (Table 2). Among these, age, thicknesses of the right lateral occipital lobe and right superior parietal lobe, volume of the caudate nucleus, Z-score, and percentage atrophy area in the VOI showed negative correlations, while the other variables showed positive correlations with MMSE scores. Variables with moderate correlation coefficients (r > 0.3) were thicknesses of the right ERC and right insular cortex, volumes of the right amygdala and right, left and total hippocampi obtained from the FreeSurfer pipeline, Z-score and percentage atrophy area in the VOI computed from VSRAD.

**Table 2:** Uni- and Multi-variate analysis between each variable and MMSE scores (N = 112).

	Univariate		Multivariate
	R square	P value	P value
R_entorhinal	0.1682	<0.0001	0.009
R_hippocampus	0.1547	<0.0001	0.406
Total hippocampus	0.1423	<0.0001	–
R_insula	0.1252	0.0001	0.083
*Z-score	0.1251	0.0001	0.087
R_amygdala	0.1188	0.0002	0.072
L_hippocampus	0.107	0.0004	0.096
*VOI (percentage of atrophy area)	0.0981	0.0008	0.084
L_amygdala	0.0852	0.0018	
L_entorhinal	0.0809	0.0024	
R_temporalpole	0.0780	0.0029	
Hippocampus/Brain	0.0734	0.0039	
R_superiortemporal	0.0727	0.0041	
Hippocampus/Cerebrum	0.0677	0.0056	
R_accumbens	0.0637	0.0073	
R_caudate	0.0581	0.0104	
L_temporalpole	0.0551	0.0128	
L_insula	0.0476	0.0208	
R_lateraloccipital	0.0421	0.0301	
L_inferiortemporal	0.0416	0.0309	
L_superiortemporal	0.0398	0.0349	
Age	0.0370	0.0423	
R_superiorparietal	0.0355	0.0466	

**Note**. Asterisks indicate variables obtained from VSRAD.

### 2.9 Univariate analysis between HDS-R and variables

Volumes of the right, left and total hippocampus, right and left amygdala, right nucleus accumbens, ratio of the hippocampus to cerebral volume, cortical thicknesses of the right and left ERC, right and left insula, right and left temporal pole, right and left inferior temporal lobes, right and left middle temporal lobes, and right and left superior temporal lobes from FreeSurfer, Z-score and percentage atrophy area in the VOI from VSRAD correlated significantly with HDS-R scores (Table 3). Among these, Z-score and percentage atrophy area in the VOI showed negative correlations, while the volume variables correlated positively with HDS-R scores. Variables with moderate correlation coefficients (r > 0.3) were right and left ERC thicknesses, cortical thicknesses of the right insula and left superior temporal lobe, volumes of the right amygdala and right and total hippocampi from the FreeSurfer pipeline, Z-score and percentage atrophy area in the VOI computed from VSRAD.

**Table 3:** Uni- and Multi-variate analysis between each variable and HDS-R scores (N = 98).

	Univariate		Multivariate
	R square	P value	P value
R_entorhinal	0.1745	<0.0001	0.044
*Z-score	0.1492	<0.0001	0.561
R_insula	0.1481	<0.0001	0.052
*VOI (percentage of atrophy area)	0.1361	0.0002	0.763
R_hippocampus	0.1295	0.0003	0.936
Total hippocampus	0.1133	0.0007	0.511
L_entorhinal	0.1116	0.0008	0.959
L_superiortemporal	0.105	0.0011	0.648
R_amygdala	0.0977	0.0017	0.104
R_temporalpole	0.0894	0.0028	
R_superiortemporal	0.0845	0.0037	
L_temporalpole	0.0829	0.0041	
L_hippocampus	0.0818	0.0043	
L_inferiortemporal	0.0807	0.0046	
L_Amygdala	0.0740	0.0067	
Hippocampus/Brain	0.0734	0.007	
Hippocampus/Cerebrum	0.0677	0.0093	
L_insula	0.0617	0.0137	
R_accumbens	0.0590	0.016	
L_middletemporal	0.0484	0.0295	
R_middletemporal	0.0441	0.0381	
R_inferiortemporal	0.0428	0.0409	
L_thalamus	0.0406	0.0466	

**Note**. Asterisks indicate variables obtained from VSRAD.

### 2.10 Multivariate logistic regression analysis between MMSE and HDS-R with independent variables

Multivariate logistic regression analysis showed only right ERC thickness correlated significantly with both MMSE score (p = 0.009) and HDS-R score (p = 0.044). Table 2, Table 3 indicate correlations of right ERC thickness with MMSE and HDS-R scores.

## 3 Discussion

In the present study, thickness of the right ERC showed significant positive correlations with both MMSE and HDS-R scores using multivariate logistic regression analysis. We also found that Z-score derived from VSRAD correlated negatively with thickness of bilateral ERCs, ratio of the hippocampus to cerebral volume, and left amygdala volume using multivariate logistic regression analysis.

The ERC is implicated in working memory and spatial information [[14], [15], [16]], communicating interactions of memory consolidation between the hippocampus and neocortex [15]. ERC volume [17] and thickness [18,19] are reportedly associated with progression of AD. Grid cells are spatially modulated neurons that have been identified in and around the ERC in mammalian species [20]. These cells play important roles in spatial cognition [16,21]. ERC may thus play a crucial role in spatial orientation and memory. MMSE scores have been widely administered to evaluate cognitive function. Moreover, the domain of spatial orientation and memory contributes to high scores in the MMSE [22]. HDS-R evaluates orientation, memory, general information, calculation, and memory recall [23]. Accordingly, our results underscore the evidence that thickness of the ERC estimates MMSE and HDS-R scores.

In our study, both MMSE and HDS-R scores were significantly associated with right ERC thickness, but not left ERC thickness. Previous research has shown that right ERC volume was larger than left ERC volume in both normal subjects and patients with AD [17]. Right ERC volume was significantly associated with progression of mild cognitive impairment to AD [7]. The apolipoprotein E (ApoE) ε4 allele is a well-established risk factor for AD. Juottonen et al. revealed that the ApoE ε4 allele contributes to atrophy, especially of the right ERC [17]. Their study also showed associations between the ApoE ε4 allele and neuropathological findings such as increases of amyloid plaques and neurofibrillary tangles. Taken together, the right ERC may play an important role in cognitive function.

Z-score derived from VSRAD correlated negatively with thickness of bilateral ERCs, ratio of the hippocampus to cerebral volume, and left amygdala volume in our study. According to previous studies [24,25], the target VOI of VSRAD is placed in medial temporal structures including the hippocampus and amygdala, as well as the ERC. As a consequence, Z-score correlated less with MMSE and HDS-R scores than thickness of the right ERC in this study and our results are in line with the targeted VOI settings on VSRAD.

Our study shows several limitations. First, handedness data were unavailable, which might have influenced laterality results. Second, some subjects underwent only one of MMSE or HDS-R. Finally, healthy individuals were not included in this study. Our results may be applicable only to patients with suspected cognitive impairment. Validation using other datasets would strengthen our results and represents the next step in our research.

In conclusion, right ERC thickness appears to offer a useful predictive biomarker for both MMSE and HDS-R scores.

## Ethical approval

This retrospective study was approved by National Hospital Organization Kyushu Medical Center Institutional Review Board for Clinical Research.

## Informed consent

Informed consent was waived because this study was retrospective nature. Author contribution

Koji Yamashita: Guarantor of integrity of the entire study, Manuscript preparation, Literature research, Experimental studies / data analysis, Statistical analysis.

Takahiro Kuwashiro: Clinical studies, Manuscript editing.

Kensuke Ishikawa: Clinical studies, Manuscript editing.

Kiyomi Furuya: Literature research.

Shino Harada: Literature research.

Seitaro Shin: Literature research.

Noriaki Wada: Literature research.

Chika Hirakawa: Data analysis.

Yasushi Okada: Manuscript editing.

Tomoyuki Noguchi: Study concepts and design, Manuscript editing.

## Funding

This work was supported by Grant of The Clinical Research Promotion Foundation.

## Declaration of Competing Interest

The authors declare that they have no conflict of interest.
